# Biological subphenotypes of acute respiratory distress syndrome may not reflect differences in alveolar inflammation

**DOI:** 10.14814/phy2.14693

**Published:** 2021-02-06

**Authors:** Nanon F. L. Heijnen, Laura A. Hagens, Marry R. Smit, Marcus J. Schultz, Tom van der Poll, Ronny M. Schnabel, Iwan C. C. van der Horst, Robert P. Dickson, Dennis C. J. J. Bergmans, Lieuwe D. J. Bos, the BASIC consortium

**Affiliations:** ^1^ Department of Intensive Care Maastricht University Medical Center+ Maastricht The Netherlands; ^2^ Department of Intensive Care Amsterdam University Medical Centers Location Academic Medical Center Amsterdam The Netherlands; ^3^ Laboratory of Experimental Intensive Care and Anesthesiology (L·E·I·C·A) Academic Medical Centers Location Academic Medical Center University of Amsterdam Amsterdam The Netherlands; ^4^ Mahidol‐Oxford Tropical Medicine Research Unit (MORU) Mahidol University Bangkok Thailand; ^5^ Nuffield Department of Medicine University of Oxford Oxford UK; ^6^ Center for Experimental and Molecular Medicine Amsterdam University Medical Centers Location Academic Medical Center University of Amsterdam Amsterdam The Netherlands; ^7^ Division of Infectious Diseases Amsterdam University Medical Centers Location Academic Medical Center University of Amsterdam Amsterdam The Netherlands; ^8^ Division of Pulmonary and Critical Care Medicine Department of Internal Medicine University of Michigan Medical School Ann Arbor MI USA; ^9^ Department of Microbiology and Immunology University of Michigan Medical School Ann Arbor MI USA; ^10^ Michigan Center for Integrative Research in Critical Care Ann Arbor MI USA

## Abstract

Biological subphenotypes have been identified in acute respiratory distress syndrome (ARDS) based on two parsimonious models: the “uninflamed” and “reactive” subphenotype (cluster‐model) and “hypo‐inflammatory” and “hyper‐inflammatory” (latent class analysis (LCA) model). The distinction between the subphenotypes is mainly driven by inflammatory and coagulation markers in plasma. However, systemic inflammation is not specific for ARDS and it is unknown whether these subphenotypes also reflect differences in the alveolar compartment. Alveolar inflammation and dysbiosis of the lung microbiome have shown to be important mediators in the development of lung injury. This study aimed to determine whether the “reactive” or “hyper‐inflammatory” biological subphenotype also had higher concentrations of inflammatory mediators and enrichment of gut‐associated bacteria in the lung. Levels of alveolar inflammatory mediators myeloperoxidase (MPO), surfactant protein D (SPD), interleukin (IL)‐1b, IL‐6, IL‐10, IL‐8, interferon gamma (IFN‐ƴ), and tumor necrosis factor‐alpha (TNFα) were determined in the mini‐BAL fluid. Key features of the lung microbiome were measured: bacterial burden (16S rRNA gene copies/ml), community diversity (Shannon Diversity Index), and community composition. No statistically significant differences between the “uninflamed” and “reactive” ARDS subphenotypes were found in a selected set of alveolar inflammatory mediators and key features of the lung microbiome. LCA‐derived subphenotypes and stratification based on cause of ARDS (direct vs. indirect) showed similar profiles, suggesting that current subphenotypes may not reflect the alveolar host response. It is important for future research to elucidate the pulmonary biology within each subphenotype properly, which is arguably a target for intervention.

## Graphical abstract

Preliminary results suggest that biological subphenotypes of ARDS may not reflect differences in alveolar inflammation and key features of the lung microbiome.

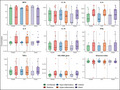

## 1 INTRODUCTION

Acute respiratory distress syndrome (ARDS) is one of the leading causes of acute respiratory failure and results in considerable morbidity and mortality (Thompson et al., 2017). So far, randomized clinical trials that investigated pharmacological interventions in an unselected population of ARDS patients did not show consistent beneficial effects in favor of the tested treatment (Matthay et al., 2017). A possible reason for the failed drug discovery for ARDS is the biological heterogeneity of the syndrome, as no singular underlying pathophysiologic mechanism is present in all patients.

Major progress has been made in splitting ARDS into subphenotypes. Previously proven unsupervised algorithms, informed by the biological heterogeneity of ARDS, split patients into two groups: a cluster‐based method resulted in the distinction of an “uninflamed” and “reactive” subphenotype (Bos et al., 2017), while latent class analysis (LCA) revealed a “hypo‐inflammatory” and “hyper‐inflammatory” subphenotype (Calfee et al., 2014). Notably, LCA‐derived subphenotypes showed a differential response to positive end‐expiratory pressure, fluid management, and simvastatin (Calfee et al., 2014, 2018; Famous et al., 2017).

The distinction between the subphenotypes is mainly driven by inflammatory and coagulation markers in plasma (Bos et al., (2017); Sinha et al., 2020). However, systemic inflammation is not specific for ARDS and it is unknown whether these subphenotypes also reflect differences in the alveolar compartment (Zador et al., 2020). Alveolar inflammation and dysbiosis of the lung microbiome have shown to be important mediators in the development of lung injury (Dickson et al., 2020; Kitsios et al., 2020; Thompson et al., 2017). Specifically, the presence of Enterobacteriaceae in the lung was predictive for both poor ICU‐outcome and the clinical diagnosis of ARDS (Dickson et al., 2020). Whether this is also captured by the defined biological subphenotypes needs to be elucidated.

We hypothesized that patients with the “reactive” or “hyper‐inflammatory” subphenotype also had higher concentrations of inflammatory mediators and enrichment of gut‐associated bacteria in the lung. Some of the results have been previously reported in the form of an abstract.

## 2 METHODS

### 2.1 Design and selection of patients

This study was a secondary analysis of the BASIC‐study (Dickson et al., 2020), a prospective observational study into biomarker analysis in septic ICU patients performed at the mixed ICU of one university‐based tertiary care hospital (Amsterdam University Medical Centers, Location Academic Medical Center in Amsterdam). They included both patients with and without ARDS between September 2011 and November 2013, of which a subset of patients was included in this analysis. Specifically, all patients of whom the representative gave permission for distant airway sampling (IRB no. NL34294.018.10) and met the following criteria: (1) mechanically ventilated, (2) diagnosed with ARDS according to the Berlin definition (ARDS Definition Task Force et al., 2012), (3) blood sample collected within 24 h of ICU admission for biological phenotyping, and (4) a miniature‐bronchoalveolar lavage (mini‐BAL) obtained within 48 h of ICU admission. Sepsis was defined as follows: (1) an infection with a probable or definite likelihood diagnosed within 24 h after ICU‐admission combined with (2) at least one parameter as described in the 2001 International Sepsis Definitions. A four‐point scale classification system derived from the CDC criteria was used to assess the plausibility of infection for every admitted patient, ascending from none, possible, probable to definite (Klein Klouwenberg et al., 2013; Vught et al., 2017).

### 2.2 Classification of subphenotypes

Biological subphenotypes were distinguished in plasma based on two previously described parsimonious models. First, a cluster‐based model distinguishing an “uninflamed” and “reactive” subphenotype based on plasma levels of interleukin‐6 (IL‐6), interferon gamma (IFN‐ƴ), angiopoietin 2/1 (ANG 1 and 2), and plasminogen activator inhibitor‐1 (PAI‐1) (Bos et al., 2017). Second, a latent class analysis (LCA) model revealing a “hypo‐inflammatory” and “hyper‐inflammatory” subphenotype using plasma levels of interleukin‐8 (IL‐8), protein C, and bicarbonate (model 3 as described by Sinha et al. (2020)). Plasma levels of IL‐6, IL‐8, IFN‐y were determined using cytometric bread analysis (Flex Set multiplex assay, BD Biosciences, San Jose, CA, USA) and levels of PAI‐1, protein C, ANG 1, and 2 by Luminex (Bio‐Rad, Hercules, California, USA) according to the manufacturer's instructions. Direct versus indirect ARDS was determined by the study investigators based on the patients’ medical record, if patients had any pulmonary insult such as pneumonia, aspiration, smoke inhalation or near‐drowning they were labeled as “direct” ARDS, irrespective of the presence of a co‐occurring non‐pulmonary risk factor such as sepsis.

### 2.3 Miniature‐bronchoalveolar lavage collection and processing

Mini‐BAL specimens were collected by a trained medical team using the standard clinical protocol. In short, as described before, specimens were collected by introducing a 50 cm 14 Fr tracheal suction catheter through the orotracheal tube. The catheter was inserted until significant resistance was encountered and subsequently pulled back 1 cm. Thereafter, 20 ml of 0.9% saline was injected in 10 seconds and immediately aspirated, after which the catheter was removed. The collected specimens were stored on ice until processing (Dickson et al., 2020). Levels of alveolar inflammatory mediators myeloperoxidase (MPO), surfactant protein D (SPD), IL‐1b, IL‐6, IL‐10, IL‐8, IFN‐ƴ, and tumor necrosis factor‐alpha (TNFα) were determined in the mini‐BAL fluid using cytometric bead analysis (Flex Set multiplex assay, BD Biosciences, San Jose, California, USA) or Luminex (Bio‐Rad, Hercules, California, USA). All analyses were run in duplicate per sample. Values below the lowest level of quantification (LLQ) were imputed with the LLQ for that biomarker. In addition, mini‐BAL fluid protein levels were corrected for dilution using the urea dilution correction method (Rennard et al., 1985):

$$\mathrm{Corrected}\mathrm{BALF}\mathrm{protein}=\mathrm{BALF}\left[x\right]\times \left(\frac{\mathrm{Urea}\mathrm{plasma}}{\mathrm{Urea}\mathrm{BALF}}\right)$$

Key features of the lung microbiome were measured according to previously published protocols (Caporaso et al., 2011; Dickson et al., 2020; Kozich et al., 2013; Mason et al., 2012): bacterial burden (16S rRNA gene copies/ml), community diversity (Shannon Diversity Index), and community composition. In short, all specimens were centrifuged (15 *g* for 15 min at 4°C) to separate the cells. Cell‐free supernatant was frozen at −80°C for subsequent assays. These cell‐free supernatants were subsequently centrifuged (22,500 *g* for 30 min), and the resulting pellet was used for DNA isolation. Bacterial DNA was isolated and extracted from mini‐BAL pellets (Qiagen DNeasy Blood & Tissue Kit, Qiagen, Hilden, Germany) and sequenced using the Illumina MiSeq platform (San Diego, CA). Bacterial DNA was quantified using a QX200 Droplet Digital PCR system (Bio‐Rad, Hercules, CA), of which two replicates were used per sample. No‐template control specimens were used which were run alongside mini‐BAL specimens. Sequence data were used to generate operational taxonomic units (OTUs) with mothur software v.1.39.5. The mothur implementation of the Ribosomal Database Project (RDP) Classifier and the RDP taxonomy training set 14 (Trainset14_030215.rdp) were used for the classification of OTUs. Sequences are available via the NCBI Sequence Read Archive. A detailed description is provided by Dickson et al. (2020).

### 2.4 Statistical analysis

Differences between groups were tested with Student's *t* test for continuous, normally distributed data and with the Mann–Whitney *U* test for continuous, non‐normally distributed data. Categorical data were tested with the Fisher‐exact test. At patient level, the ratio of pulmonary inflammatory mediators versus systemic inflammatory mediators was calculated by:

Inflammatorymediatorratio=log2mediatoralveolarureaalveolarmediatorsystemicplasmaurea systemicplasma

The lung microbiome bacterial communities composition was assessed using principal coordinate (PCO) analysis. A set of 135 bacterial families was projected onto PCOs to reduce the dimensionality, of which the first two PCOs explained, respectively, 21% and 14% of the variance. Tukey's “Honest Significant Difference” (“stats” package) was used to compare means between the groups. Next, a rank abundance analysis was performed to identify enriched families followed by a random forest analysis to identify discriminating taxonomic groups (“mvabund,” “vegan,” and “randomforest” package in R). Each analysis was performed for (1) direct versus indirect ARDS, (2) cluster‐derived subphenotypes, and (3) LCA‐derived subphenotypes to ensure consistency regardless of the chosen phenotyping method. A *p*‐value of 0.05 was considered statistically significant. All analyses were performed in R version 3.6.2 using the R‐studio interface.

## 3 RESULTS

**FIGURE 1:**
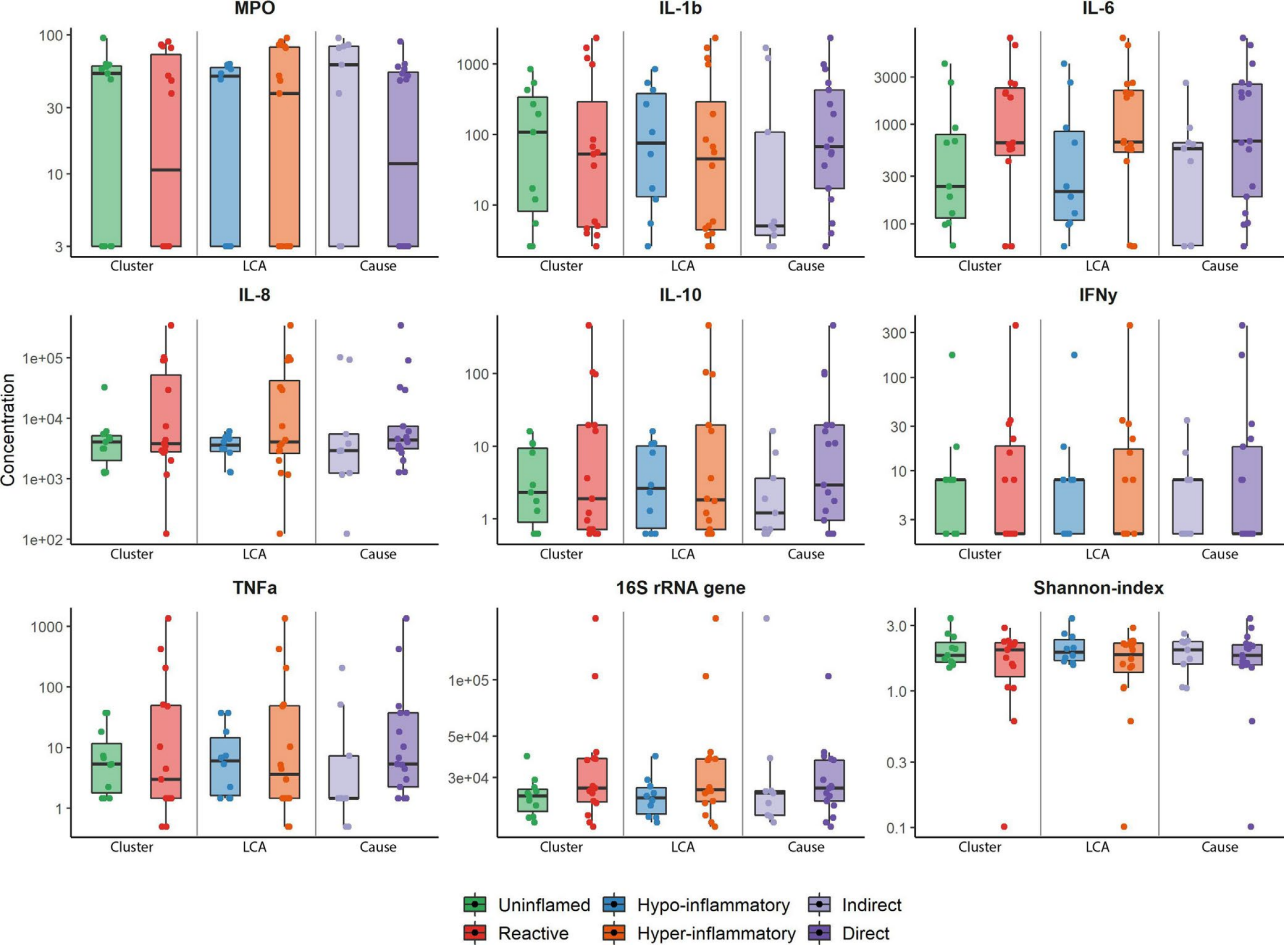
Alveolar inflammation per ARDS (sub)phenotype. Subscription: Boxplots indicate mean values with inter‐quartile range and 95% range. Individual datapoints are also shown. Cluster subphenotypes are depicted in green for uninflamed and red for reactive. LCA subphenotypes are illustrated in blue for hypo‐inflammatory and orange for hyper‐inflammatory. Indirect ARDS is shown in light purple and direct ARDS in darker purple. For all comparisons, the p‐value was >0.1, without correction for multiple testing. Only uncorrected concentrations are depicted. Abbreviations: Myeloperoxidase (MPO) ng/ml, interleukin 1 beta (IL‐1b) pg/ml, interleukin 6 (IL‐6) pg/ml, interleukin 8 (IL‐8) pg/ml, interferon gamma (IFN‐ƴ) pg/ml, tumor necrosis factor‐alpha (TNFα) pg/ml, 16S rRNA genes copies/ml

**FIGURE 2:**
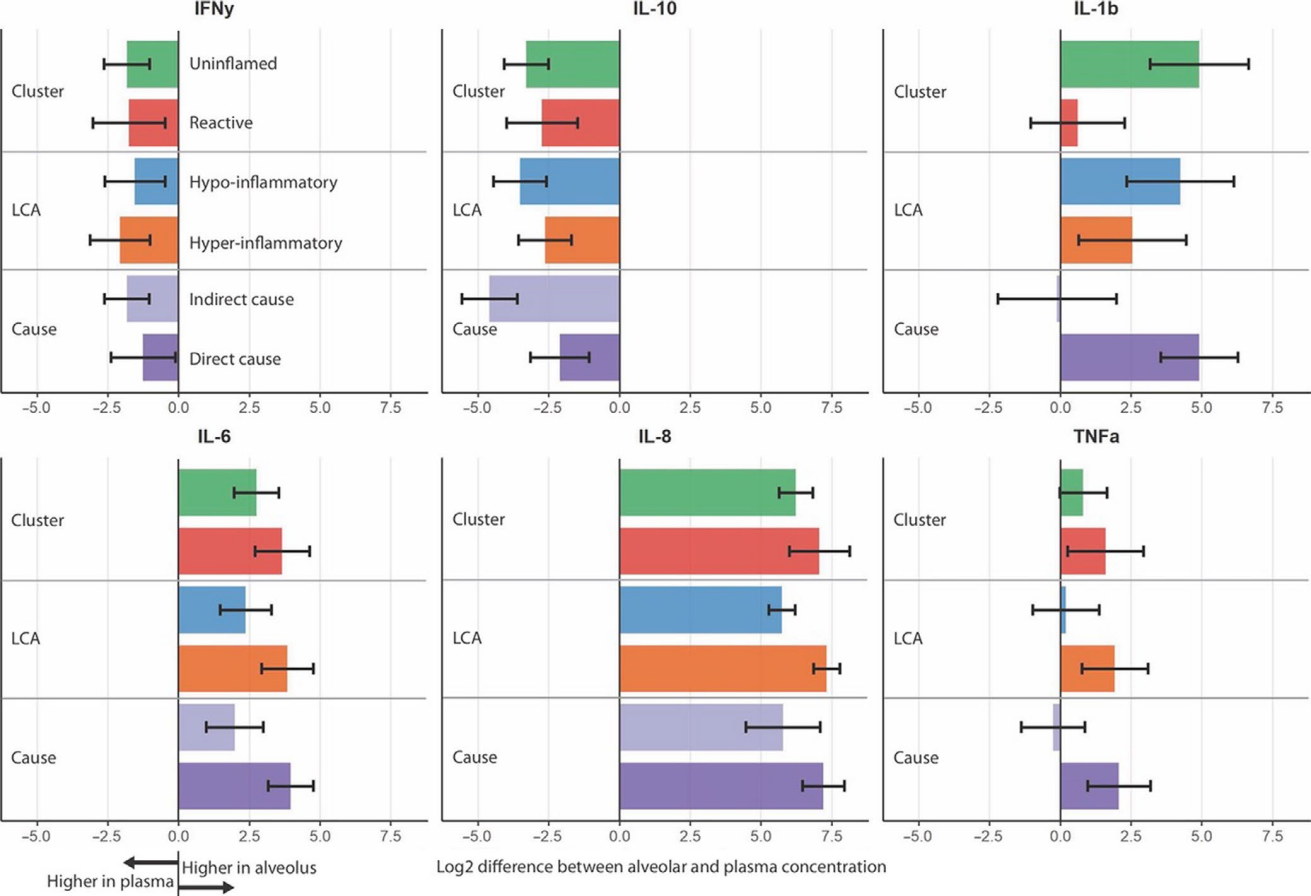
Log2 difference between alveolar and plasma concentration per (sub)phenotype. Subscription: Bars indicate mean values with a standard error of the mean. Positive values indicate a higher alveolar concentration, while a negative value indicates a higher plasma level of the biomarker. Cluster subphenotypes are illustrated in green for uninflamed and red for reactive. LCA subphenotypes are depicted in blue for hypo‐inflammatory and orange for hyper‐inflammatory. Indirect ARDS is shown in light purple and direct ARDS in darker purple. For all comparisons, the *p*‐value was >0.1, without correction for multiple testing. Abbreviations: Interferon gamma (IFN‐ƴ) pg/ml, interleukin 1 beta (IL‐1b) pg/ml, interleukin 6 (IL‐6) pg/ml, interleukin 8 (IL‐8) pg/ml, and tumor necrosis factor‐alpha (TNFα) pg/ml

Twenty‐six patients were included in the analysis, of whom 15 (58%) had a cluster‐derived “reactive” subphenotype and 11 (42%) had an “uninflamed” subphenotype (Table 1). The included patients were representative of the ARDS population in the original cohort (Bos et al., 2017) with an average age of 60 years (SD ±13), an APACHE IV score of 91 (SD ±28), and pneumonia as the predominant risk factor for ARDS (61.5%). Patients with a “reactive” subphenotype had a higher sequential organ failure assessment score on day 1 (SOFA score, mean: 11, SD ±2.9) compared to the “uninflamed” subphenotype (mean: 7, SD ±2.8, *p* = 0.007), in line with previous reports (Bos et al., 2017). No statistically significant differences between the “uninflamed” and “reactive” ARDS subphenotypes were found in alveolar inflammatory mediators and key features of the lung microbiome (Table 2; Figure 1). These results did not change after correction for the urea dilution factor (Table 2). The alveolar‐systemic inflammatory mediator ratios were not significantly different between cluster phenotypes. Noteworthy, the inflammatory mediators were present in much higher concentrations in the alveolar compartment than in the systemic compartment, with the exception of IFNy and IL‐10 as depicted by their negative alveolar‐systemic inflammatory mediator ratios (Figure 2).

**TABLE 1:** Patient demographics and clinical characteristics per (sub)phenotype

	Cluster subphenotypes			LCA subphenotypes			Etiology phenotype		
	Uninflamed	Reactive	*p*‐value	Hypo‐inflammatory	Hyper‐inflammatory	*p*‐value	Indirect	Direct	*p*‐value
	*n* = 11	*n* = 15		*n* = 10	*n* = 16		*n* = 9	*n* = 17	
Age in years (mean (±SD))	58 (13)	62 (13)	0.50	61 (14)	60 (12)	0.92	59 (12)	61 (13.6)	0.65
Female (*n* (%))	5 (45.5)	8 (53.3)	1.00	3 (30.0)	10 (62.5)	0.23	6 (66.7)	7 (41.2)	0.41
APACHE IV (mean (±SD))	82 (24.3)	97 (29.8)	0.18	80 (22.2)	98 (30.1)	0.13	93 (33.6)	89.5 (25.9)	0.75
SOFA score at day 1 (mean (±SD))	7 (2.8)	11 (2.9)	0.007	7.4 (3.1)	10.4 (2.8)	0.02	10.6 (3.1)	8.6 (3.2)	0.15
ARDS risk factor (*n* (%))									
Pneumonia	8 (72.7)	8 (53.3)	0.43	8 (80.0)	8 (50.0)	0.22	0 (0.0)	16 (94.1)	<0.001
Aspiration	1 (9.1)	1 (6.7)	1.00	1 (10.0)	1 (6.2)	1.00	0 (0.0)	2 (11.8)	0.53
Sepsis	8 (72.7)	14 (93.3)	0.28	7 (70.0)	15 (93.8)	0.26	7 (77.8)	15 (88.2)	0.59
Trauma	2 (18.2)	0 (0.0)	0.17	2 (20.0)	0 (0.0)	0.14	1 (11.1)	1 (5.9)	1.00
Pancreatitis	0 (0.0)	1 (6.7)	1.00	0 (0.0)	1 (6.2)	1.00	1 (11.1)	0 (0.0)	0.35
Other	0 (0.0)	0 (0.0)		0 (0.0)	0 (0.0)		0 (0.0)	0 (0.0)	
ARDS severity^a^ (*n* (%))									
Mild	6 (54.5)	2 (13.3)	0.06	6 (60.0)	2 (12.5)	0.03	3 (33.3)	5 (29.4)	0.56
Moderate	4 (36.4)	12 (80.0)		4 (40.0)	12 (75.0)		6 (66.7)	10 (58.8)	
Severe	1 (9.1)	1 (6.7)		0 (0.0)	2 (12.5)		0 (0.0)	2 (11.8)	
Ventilator‐free days (median (IQR))	24.0 (3.5, 25.5)	19.0 (7.5, 21.5)	0.19	24.5 (1.8, 25.8)	19.5 (11.3, 22.3)	0.29	21.0 (17,0, 24.0)	19.0 (0.0, 25.0)	0.70
ICU length of stay (median (IQR))	6.0 (3.5, 11.0)	8.0 (6.5, 12.0)	0.48	6.5 (4.5, 8.8)	8.5 (5.3, 14.0)	0.46	9.0 (6.0, 14.0)	7.0 (4.0, 10.0)	0.48
30‐day survival (*n* (%))	8 (72.7)	9 (60.0)	0.68	7 (70.0)	10 (62.5)	1.00	6 (66.7)	11 (64.7)	1.00

Abbreviations: APACHE IV, Acute Physiology and Chronic Health Evaluation score IV; ARDS, acute respiratory distress syndrome; SOFA, Sequential Organ Failure Assessment Score on day 1.

^a^ Based on the Berlin criteria.

**TABLE 2:** Urea dilution corrected and uncorrected concentrations of inflammatory mediators in the lung and key features of the lung microbiome per (sub)phenotype

	Uncorrected			Corrected		
	Uninflamed, *n* = 11	Reactive, *n* = 15	*p*‐value	Uninflamed, *n* = 11	Reactive, *n* = 15	*p*‐value
MPO (ng/ml) (median (IQR))	53.0 (3.0, 60.0)	20.5 (3.0, 73.5)	0.57	1471.1 (117.9, 2642.1)	603.0 (296.4, 843.7)	0.23
SPD (ng/ml) (median (IQR))	36.0 (24.0, 61.5)	25.5 (8.3, 61.0)	0.58	1496.7 (404.3, 2714.7)	578.9 (63.1, 1051.0)	0.27
IL‐1b (pg/ml) (median (IQR))	108.8 (8.8, 351.3)	53.1 (4.9, 535.6)	0.94	1891.5 (436.7, 5965.0)	471.1 (197.8, 14,387.5)	0.55
IL‐6 (pg/ml) (median (IQR))	237.5 (115.2, 800.5)	654.9 (492.5, 2325.1)	0.45	7279.6 (5117.2, 28,986.2)	11164.5 (6827.8, 40,133.5)	0.29
IL‐8 (pg/ml) (median (IQR))	4048.5 (2207.9, 5131.1)	3795.3 (2776.0, 59,737.3)	0.62	132,720.3 (74,684.1, 154,140.5)	76,796.2 (34,743.1, 1,438,868.1)	0.98
IL‐10 (pg/ml) (median (IQR))	2.3 (1.0, 9.5)	1.9 (0.7, 19.7)	0.53	76.2 (36.0, 229.7)	90.3 (49.6, 318.0)	0.66
IFN‐y (pg/ml) (median (IQR))	8.0 (2.1, 8.0)	2.1 (2.1, 18.8)	0.93	163.4 (100.4, 273.7)	202.8 (36.2, 412.2)	0.98
TNFa (pg/ml) (median (IQR))	5.3 (1.9, 12.8)	3.0 (1.5, 49.3)	0.83	234.7 (69.5, 266.0)	139.6 (45.6, 550.0)	0.78
16S rRNA gene copies (median (IQR))	23,870.2 (19,853.4, 25,920.8)	26,269.9 (22,178.9, 37,850.0)	0.22			
Shannon‐index (median (IQR))	1.8 (1.6, 2.3)	2.0 (1.3, 2.2)	0.59			
Enterobacteriaceae (*n*, (%))	1 (9.1)	5 (33.3)	0.33			

	Hypo‐inflammatory, *n* = 10	Hyper‐inflammatory, *n* = 16	*p*‐value	Hypo‐inflammatory, *n* = 10	Hyper‐inflammatory, *n* = 16	*p*‐value
MPO (ng/ml) (median (IQR))	50.5 (3.0, 58.5)	38.0 (3.0, 82.0)	0.91	1362.3 (199.3, 2196.7)	606.0 (165.6, 922.5)	0.32
SPD (ng/ml) (median (IQR))	43.5 (7.0, 61.8)	30.0 (12.0, 59.0)	0.82	1509.0 (154.0, 2748.1)	853.4 (86.9, 1165.6)	0.54
IL‐1b (pg/ml) (median (IQR))	80.9 (13.4, 391.1)	46.3 (4.5, 393.5)	0.67	4912.2 (481.4, 5971.2)	404.9 (138.1, 13933.3)	0.23
IL‐6 (pg/ml) (median (IQR))	212.5 (108.6, 858.6)	664.9 (526.3, 2212.2)	0.32	8594.1 (5627.3, 32055.2)	9879.0 (6301.1, 39298.0)	0.64
IL‐8 (pg/ml) (median (IQR))	3589.8 (2811.5, 4771.9)	4060.3 (2631.6, 46751.3)	0.46	140580.5 (85330.5, 156466.2)	72051.2 (35658.9, 1419071.6)	0.75
IL‐10 (pg/ml) (median (IQR))	2.6 (0.8, 10.2)	1.8 (0.7, 19.6)	0.58	93.4 (52.7, 233.5)	75.0 (25.8, 311.2)	0.64
IFN‐y (pg/ml) (median (IQR))	8.0 (2.1, 8.0)	2.1 (2.1, 17.2)	0.78	201.4 (124.5, 288.3)	135.4 (33.5, 406.9)	0.32
TNFa (pg/ml) (median (IQR))	6.0 (1.7, 15.5)	3.7 (1.5, 48.5)	0.85	243.4 (123.5, 271.9)	94.8 (44.8, 420.6)	0.27
16S rRNA gene copies (median (IQR))	23284.7 (19157.0, 26421.0)	25869.6 (22325.1, 37744.9)	0.23			
Shannon‐index (median (IQR))	1.9 (1.7, 2.4)	1.9 (1.4, 2.2)	0.29			
Enterobacteriaceae (*n*, (%))	0 (0.0)	6 (37.5)	0.08			

	Indirect, *n* = 9	Direct, *n* = 17	*p*‐value	Indirect, *n* = 9	Direct, *n* = 17	*p*‐value
MPO (ng/ml) (median (IQR))	61.0 (3.0, 83.0)	25.0 (3.0, 54.0)	0.17	983.9 (379.1, 2313.4)	636.9 (69.1, 1307.8)	0.28
SPD (ng/ml) (median (IQR))	35.0 (9.0, 63.0)	33.0 (17.0, 56.8)	0.96	1100.4 (72.8, 1496.7)	660.2 (90.8, 2681.3)	0.91
IL‐1b (pg/ml) (median (IQR))	5.1 (3.7, 108.8)	67.4 (17.4, 430.8)	0.17	338.8 (110.8, 4125.0)	1891.5 (424.5, 13479.2)	0.27
IL‐6 (pg/ml) (median (IQR))	571.4 (60.4, 654.9)	674.9 (187.4, 2550.9)	0.17	7301.0 (5293.9, 35124.3)	9908.6 (6510.9, 36324.2)	0.37
IL‐8 (pg/ml) (median (IQR))	2902.7 (1246.0, 5418.8)	4325.3 (3119.7, 7393.3)	0.32	67306.1 (32911.6, 479589.5)	132720.3 (68354.7, 291031.9)	0.61
IL‐10 (pg/ml) (median (IQR))	1.2 (0.7, 3.6)	3.0 (1.0, 19.6)	0.22	58.0 (26.6, 90.3)	124.2 (48.0, 331.6)	0.11
IFN‐y (pg/ml) (median (IQR))	8.0 (2.1, 8.0)	2.1 (2.1, 18.0)	1.00	302.8 (90.4, 401.6)	163.4 (73.7, 244.7)	0.54
TNFa (pg/ml) (median (IQR))	1.5 (1.5, 7.3)	5.3 (2.2, 37.1)	0.12	62.6 (28.6, 277.8)	188.5 (76.8, 291.1)	0.27
16S rRNA gene copies (median (IQR))	24740.0 (18798.0, 25469.3)	26269.9 (22471.4, 37108.5)	0.57			
Shannon‐index (median (IQR))	2.0 (1.6, 2.3)	1.8 (1.6, 2.2)	0.77			
Enterobacteriaceae (*n*, (%))	2 (22.2)	4 (23.5)	1.00			

Abbreviations: myeloperoxidase (MPO), surfactant protein D (SPD), interleukin (IL)‐1 beta, ‐6, ‐8, ‐10, interferon gamma (IFN‐y), tumor necrosis factor alpha (TNFa), and 16S rRNA genes (16s). Corrected BALF protein levels as calculated by: "Corrected BALF protein = (BALF (X)) × (urea plasma)/(urea BALF)".

Stratification based on the cause of ARDS (direct vs. indirect) and subphenotypes based on the parsimonious model that predicts LCA‐derived subphenotypes showed similar results to cluster‐derived subphenotypes with respect to all inflammatory mediators (Table 2; Figures 1 and 2). Furthermore, *Enterobacteriaceae* spp. were found in the mini‐BAL fluid of 6/16 patients with the LCA‐derived “hyper‐inflammatory” subphenotype, but in none of the patients with the “hypo‐inflammatory” subphenotype. However, this did not reach statistical significance (*p* = 0.08).

**FIGURE 3:**
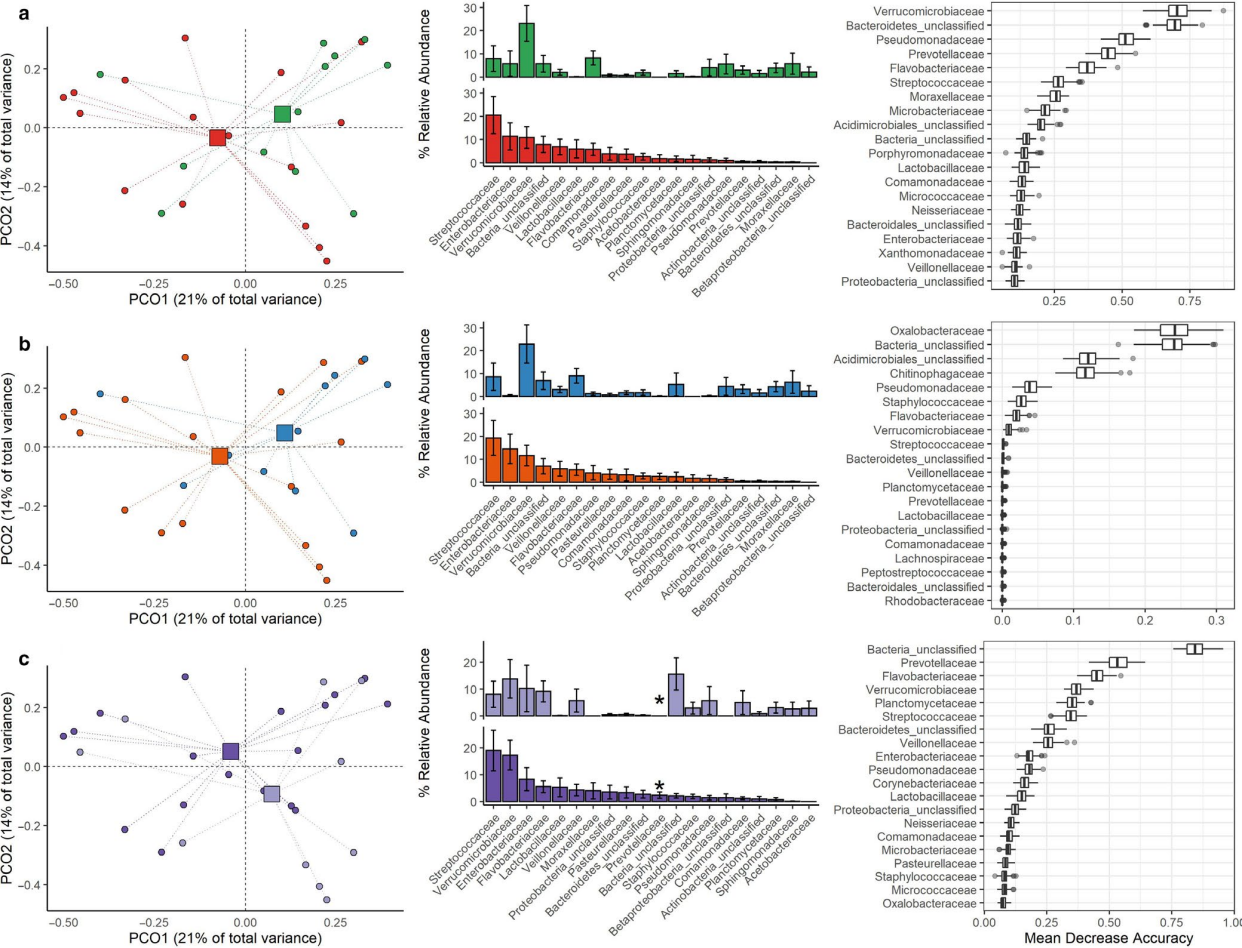
In‐depth analysis of the lung microbiome per ARDS (sub)phenotype. Subscription: Left‐to‐right: Principal coordinate (PCO) analysis of bacterial communities, rank abundance analysis, and random forest analysis. (a) cluster‐subphenotype: reactive (green) and uninflamed (red). (b) LCA‐subphenotypes: hypo‐inflammatory (blue) and hyper‐inflammatory (orange). (c) etiology phenotype: Indirect (light purple) and Direct (dark purple). Bacterial communities did not significantly differ for individual subphenotypes (PCO). Rank abundance analysis only showed enriched *Prevotellaceae* in patients with “direct” ARDS. However, random forest analysis did not confirm this family as a discriminating taxonomic group

Exploratory in‐dept analysis of the lung microbiome by principal coordinate analysis of the bacterial community composition of all 28 patients revealed that compositions clustered together, irrespective of the subphenotyping method. This was reflected by a non‐significant difference between the subphenotyping methods and PCO1 and PCO2 (all p‐values above 0.5) (Figure 3). Moreover, rank abundance analysis (LCA‐subphenotype: % relative abundance Enterobacteriaceae spp. *p* = 0.89) and random forest analysis did not identify enriched taxa.

## 4 DISCUSSION

This study presents the first comparison between systemic biological subphenotypes of ARDS and alveolar inflammation and dysbiosis of the lung microbiome. The results can be summarized as follows: (1) patients with the cluster‐derived “reactive” subphenotype do not have higher concentrations of a selected set of inflammatory mediators in the lung, (2) the inflammatory mediators are higher in the alveolar compartment in all subgroups, (3) these results are largely confirmed in the LCA‐derived subphenotypes, although (4) there was a trend toward the more frequent presence of Enterobacteriaceae spp. in the LCA‐derived “hyper‐inflammatory” subphenotype, despite the similar bacterial community composition of the lung microbiome, that deserves further attention.

In this study, we unraveled whether the subphenotypes that are considered more inflammatory (“reactive” and “hyper‐inflammatory”) based on plasma biomarkers also show higher concentrations of inflammatory mediators in the alveolar compartment and concluded that there are no profound differences. Although this particular question has not been addressed previously, our results are in line with a vast body of literature that directly compared inflammatory cytokines in plasma and BAL‐fluid. Although ARDS is associated with elevated levels of pro‐inflammatory cytokines in both compartments, no relationships were found between levels of singular inflammatory cytokines in BAL‐fluid and plasma (Binnie et al., 2014; Meduri, Headley, et al., 1995; Meduri, Kohler, et al., 1995; Park et al., 2001; Pittet et al., 1997).

Under normal circumstances, the alveolar and systemic compartments are separated by a strong barrier that limits the free movement of inflammatory cytokines, and other proteins, across the basement membrane. Lung injury affects the barrier function resulting in a capillary leak. Therefore, as hypothesized, it could be speculated that the observed higher concentrations of inflammatory cytokines in plasma in the “reactive” and “hyper‐inflammatory” subphenotypes, result in higher alveolar concentrations as well. However, this is not what we observed. The alveolar concentration of many of the inflammatory mediators that we investigated was higher than in plasma, when corrected for urea concentration differences that reflect the dilution of the BAL fluid (urea is a metabolite that can freely equilibrate between compartments, also under normal circumstances). Furthermore, the lack of differences in the BAL concentration of IL‐8 and MPO between the subphenotypes and the widespread within each subphenotype could indicate that there is considerable heterogeneity in neutrophil activation in the lung that is not accounted for by the determination of systemic biological subphenotypes. The alveolar/systemic discrepancy we found is in line with the recent findings of Morrell et al. They described that alveolar macrophages and blood monocytes show distinct gene expression profiles and that the gene expression profile predicting the prolonged duration of mechanical ventilation differed between the two cell populations (Morrell et al., 2018). Taken together, these observations may suggest that the currently known subphenotypes do not reflect the alveolar host response, but that the alveolar space is the site of profound inflammation in ARDS.

Our results also showed a trend toward the more frequent presence of *Enterobacteriaceae* spp. in LCA‐derived “hyper‐inflammatory” subphenotype (despite the absence of additional findings in the in‐depth analysis). It is well known that the lung microbiome of critically ill patients is altered compared to healthy persons (Dickson et al., 2016; Panzer et al., 2018; Zakharkina et al., 2017). Acute respiratory distress syndrome is associated with enrichment of the lung microbiome with gut‐associated *Enterobacteriaceae* spp. (Dickson et al., 2020; Panzer et al., 2018). Previous studies showed that the lung microbiome composition of critically ill patients is not only a predictor for ARDS development and ICU‐outcome, but is also associated with alveolar and systemic inflammation (Dickson et al., 2016, 2020; Kitsios et al., 2020; Panzer et al., 2018). Panzer et al. found that the lung microbial composition at admission was related to plasma intercellular adhesion molecule‐1, vascular endothelial growth factor, and IL‐8 and that the variation 48 h after hospitalization was associated with IL‐6 and IL‐8. They also observed that the relationship between these inflammatory parameters and the microbiome composition was driven by the presence or absence of specific taxa, suggesting that specific bacteria can contribute to inflammation at certain timepoints during the course of the disease/syndrome (Panzer et al., 2018). Another study revealed that the presence of gut‐associated *Bacteroides* in the lung of ARDS patients was associated with concurrent serum TNFα concentration (Dickson et al., 2016). Although the precise role of the lung microbiome in acute lung injury still needs to be elucidated, the composition of the lung microbiome seems to be related to markers of endothelial‐ and epithelial injury and inflammation. It could be that the trend toward more frequent presence of *Enterobacteriaceae* spp. in the “hyper‐inflammatory” subphenotype is associated with the more systemic inflammatory profile of this subphenotype compared to the “hypo‐inflammatory” subphenotype, especially since IL‐8 is an important variable included in the biomarker set used for splitting patients into “hyper‐inflammatory” and “hypo‐inflammatory” subphenotype (Sinha et al., 2020). It would be interesting to further delve into the role of the lung microbiome in ARDS pathogenesis and additionally in the ARDS subphenotypes, as it may harbor prevention targets and new treatable traits.

There are some important limitations we need to take into account when interpreting our results. First, the limited sample size is possibly one reason for the lack of significantly different results in alveolar inflammatory mediator concentrations between direct and indirect ARDS. Since this was a secondary analysis, no sample size calculation was performed for this specifically chosen subset of patients. We intended to perform an exploratory analysis to reveal any kind of underlying signal substantiating future research, which might have resulted in type I and II errors. Furthermore, if systemic subphenotypes would be strongly reflective of alveolar inflammation, we would have identified those differences also in this small subset. In other words, there still can be a statistically significant difference in alveolar host response between the known subphenotypes, but our results make it unlikely that these differences are of such clinical importance that there is no need to further assess local inflammation in future research. Second, the timing of the mini‐BAL could have been too early. However, at diagnosis, ARDS is already associated with the enrichment of gut‐associated bacteria. This enrichment also seems to predict the outcome of the critically ill and the samples used for that analysis were taken in an even smaller time window than we managed, namely: within 24 h after admission (Dickson et al., 2020). Third, mini‐BAL was used to obtain samples from the distal airways. It is well known that mini‐BAL provides variable results, which we tried to limit by using a trained medical team, a standardized clinical sampling protocol, and correcting for dilution using the urea method. However, as mini‐BAL is a local or regional sampling method, it does not fully reflect the spatial heterogeneity seen in the lung of ARDS (Gattinoni et al., 2001). This causes both inter‐ and intra‐subject variability and could have influenced our results.

These preliminary results stress the importance of elucidating the pulmonary biology within the biological subphenotypes of ARDS. More sophisticated analysis methods and study designs are needed to unravel the pulmonary biology within the subphenotypes. This might yield information for a potential target for intervention. Future research should likely include a more in‐depth analysis of the host response (including the evolution in time) in combination with an evaluation of the enrichment of the lung microbiome for gut‐associated bacteria.

In conclusion, these preliminary results suggest that the plasma marker driven biological subphenotypes do not show profound differences in a selected set of alveolar inflammatory mediators and key features of the lung microbiome. Despite this study's considerable limitations, this emphasizes the importance of future research to elucidate the pulmonary biology within each subphenotype properly, which is arguably a target for intervention.

## AUTHOR'S CONTRIBUTIONS

All authors contributed to the study concept and design. LDJB performed the data collection. NFLH and LDJB performed the data analysis and wrote the first draft of the manuscript. All authors commented on previous versions of the manuscript. All authors read and approved the final manuscript.

## Collaborators

Published on behalf of the BASIC consortium:

BASIC consortium: Amsterdam University Medical Centers, location AMC, Amsterdam, The Netherlands: de Beer FM, Bos LD, Claushuis TA, Glas GJ, Horn J, Hoogendijk AJ, van Hooijdonk RT, Huson MA, de Jong MD, Juffermans NP, Lagrand WA, van der Poll T, Scicluna B, Schouten LR, Schultz MJ, van der Sluijs KF, Straat M, van Vught LA, Wieske L, Wiewel MA, Witteveen E.
